# Associations of levels of peripheral blood leukocyte and subtypes with type 2 diabetes: A longitudinal study of Chinese government employees

**DOI:** 10.3389/fendo.2023.1094022

**Published:** 2023-03-24

**Authors:** Ruimin Li, Ling Li, Bibo Liu, Dan Luo, Shuiyuan Xiao

**Affiliations:** Department of Social Medicine and Health Management, Xiangya School of Public Health, Central South University, Changsha, China

**Keywords:** leukocyte, leukocyte subtypes, type 2 diabetes, China, government employees

## Abstract

**Objectives:**

Available evidence suggests that type 2 diabetes (T2D) may be associated with inflammation and that leukocytes are a topical clinical, biological indicator of inflammation. This study investigates the associations between peripheral blood leukocyte and subtypes levels with T2D.

**Methods:**

A total of 5,475 individuals were included in the baseline examination from January 2018 to April 2020, with incidence data updated to April 30, 2021, and follow-up to 5,362 individuals. T2D was defined according to the Chinese guidelines for preventing and treating type 2 diabetes. Physiological and biochemical indicators, including leukocyte and subtypes, were obtained from the physical examination results of the tertiary care hospitals relied on at the cohort sites. Covariates such as demographic characteristics and lifestyle were collected by questionnaire. Binary logistic regression and Cox proportional hazard models were used to explore the correlations. Receiver Operating Characteristic (ROC) curves and time-dependent ROC curves were used to estimate the predictive diagnosis of T2D across the subtype of leukocytes.

**Results:**

The mean follow-up time was 12 months, and the cumulative incidence density of T2D was 4.0/1000 person-years. Cross-sectional results at baseline showed that the levels of peripheral blood leukocyte and its subtypes were higher in the T2D group than in the non-T2D group. Total leukocyte count and subtypes levels were grouped by quintile. After adjusting for age, sex, family history of diabetes, lifestyle score, and triglyceride levels, all were compared with the lowest quintile of each group. Logistic regression model results showed that the corrected OR for those with the highest quintile level of leukocyte was 2.01 (95% CI: 1.02-3.98). The longitudinal analysis showed that the adjusted HR was 8.43 (95%CI: 1.06-66.92) for those with the highest quintile level of leukocytes at baseline after controlling for the effects of the above covariates. For those with the highest quintile level of neutrophils at baseline, the adjusted HR was 5.05 (95%CI: 1.01-25.29). The leukocyte and subtypes had predictive values for T2D.

**Conclusion:**

Patients with T2D have a higher level of peripheral blood leukocyte and subtypes than those without the disease. Elevated leukocyte and neutrophil counts may link to a higher risk of T2D.

## 1 Introduction

According to the 9^th^ edition of the IDF Atlas of Diabetes published in 2019, diabetes has developed into a global pandemic non-communicable epidemic, with 90% of people with diabetes having T2D. In 2019, the number of people with diabetes in China exceeded 100 million, which is the highest number of people with diabetes in the world (1). Numerous previous studies have demonstrated the heavy disease burden, many complications, and poor prognosis of diabetes, which significantly negatively impact human health and social development (2–4). It is worth noting that the incidence of T2D among children and adolescents in China is also on a significant rise (5), which means that the burden of T2D and related diseases among adults in China will gradually increase. Therefore, active prevention, early diagnosis, and treatment are effective measures to control the prevalence of diabetes and improve people’s quality of life.

T2D is a multi-causal metabolic disease, with lifestyle, genetic factors, aging, obesity, oxidative stress, and inflammation as risk factors (6–9). Additionally, a mutually reinforcing relationship between cellular stress and local inflammation forms a vicious circle of inflammation (10). Previous studies have also illustrated the association between lifestyle factors such as tobacco, alcohol, diet, physical activity, and inflammation and T2D (11). Moreover, lifestyle is a modifiable risk factor and a pivotal point in our efforts to combat T2D (6, 12, 13). Leukocytes are a recognized indicator of subclinical low-grade inflammation. There is growing evidence that subclinical low-grade inflammation plays an important role in the pathogenesis of T2D (14, 15). It has been proposed that many inflammatory markers change with the progression of T2D, such as white blood cell counts, neutrophils, lymphocytes, monocytes, and C-reactive protein. Healthcare providers can determine the progression of each clinical stage of diabetes and complications based on the changes in these inflammatory markers (16, 17). Notably, evidence suggests that a high peripheral blood leukocyte count level in patients with T2D is typical of their clinical inflammation (18, 19). Leukocyte count can also predict the incidence of T2D. Leukocyte subtypes, such as elevated neutrophils and lymphocytes in peripheral blood, increase the risk of T2D in people (20, 21). Meanwhile, research evidence suggests that various anti-inflammatory drugs can treat T2D (22). Metformin, the first-line medication for treating T2D worldwide, improves chronic inflammation by improving metabolic parameters and has direct anti-inflammatory effects (23). Therefore, healthcare providers may be able to use these simple and reliable inflammatory biomarkers to screen high-risk populations and monitor clinical progress and the effectiveness of interventions. However, to our knowledge, these studies used parts of behavioral patterns or dietary habits to represent a lifestyle. Therefore, it was necessary to generate a composite indicator, including body shape, behavioral habits, and dietary habits: we created a composite score based on each participant’s smoking status, alcohol consumption, physical activity, dietary habits, and body shape. At a holistic level, the lifestyle of each participant was assessed. This newly established index was controlled as a covariate. Then, the effect of lifestyle on leukocytes could be controlled more scientifically.

In conclusion, more research evidence suggests that inflammation may be involved in the pathophysiological process of diabetes and that various anti-inflammatory drugs can be used to treat T2D. Moreover, some hypoglycemic drugs have been shown to improve chronic inflammation. However, the shortcomings of existing studies, including the need for more evidence from prospective cohort studies and poor control of the effects of numerous potential confounders, leave this evidence open to further validation. In this study, we selected a representative sample from the cohort of Chinese governmental employees. The relationship between peripheral blood leukocytes and its subtypes levels and T2D was explored, adjusting for classical diabetes risk factors: age, sex, dietary habits, lifestyle behavioral habits, body shape, and fasting triglyceride levels.

## 2 Methods

### 2.1 Study population

This study was based on a cohort study of chronic diseases in Chinese government employees conducted in four major cities in Hunan Province, China (Changsha, Zhuzhou, Xiangtan, and Huaihua). An earlier study provided a detailed description of the cohort study design (24). Briefly, a representative sample from January 2018 to April 2020 was obtained through multi-stage sampling, and incidence data were updated to April 30, 2021. First, four cities in Hunan Province were selected based on their economic development level and geographical location. Second, A random selection of governmental employee units from those that volunteered to participate in our study. Third, a whole-group sampling method was used to include all staff members of these sampled units in our study. After fully informed consent, participants filled out an electronic self-report questionnaire using electronic devices such as cell phones. Exclusion criteria: 1) Participants aged >60 years old or <18 years old at baseline, 2) Participants with missing information on lifestyle factors and other covariate questionnaires at baseline, participants who did not complete the physical examination items involved in the study, 3) Participants with cancer at baseline.

**Figure 1:**
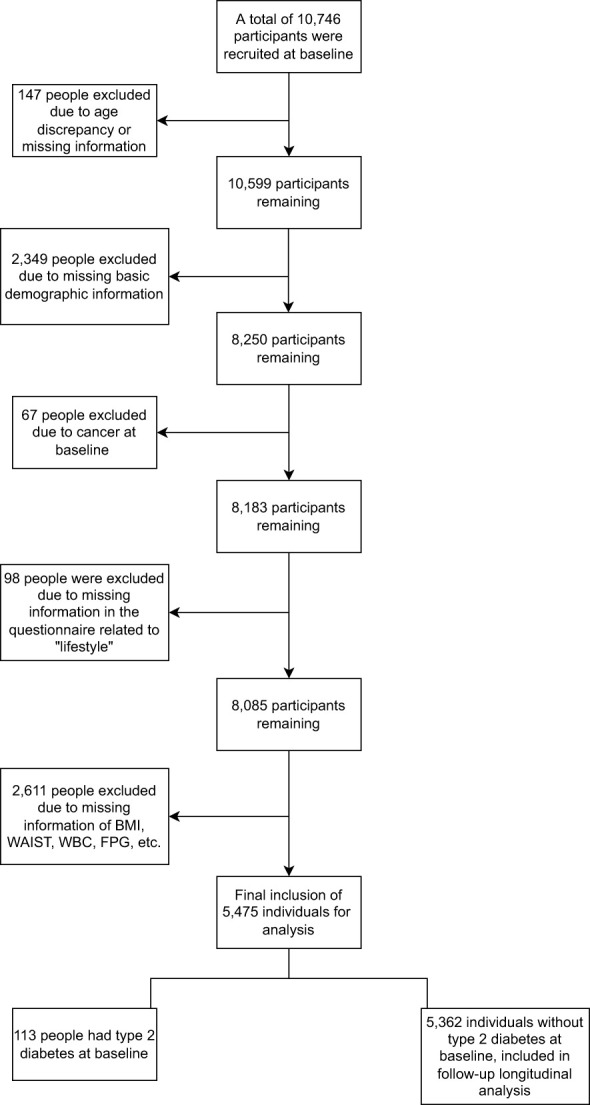
Study design flow diagram.

Accordingly, the current study included 10,746 baseline participants, with 5,271 non-eligible individuals excluded. In total, 5,475 participants were eligible for follow-up analysis (**Figure 1**). The Ethics Committee approved the study of Xiangya School of Public Health, Central South University, and informed consent was obtained from all participants.

### 2.2 Assessment of covariates

We constructed a lifestyle score to assess lifestyle factors’ impact (25). Lifestyle factors in this study consisted of three components: behavioral habits, dietary habits, and body shape; of which behavioral habits and dietary habits were obtained through a structured questionnaire and included smoking, alcohol consumption, physical activity, and diet. Non-smoking was identified as a low-risk lifestyle, defined in the questionnaire as smoking less than 100 cigarettes in a lifetime. Not drinking alcohol was identified as a low-risk lifestyle, defined in the questionnaire as consuming alcohol no more than once per week. Physical activity was defined as exercising more than once a week for a low-risk lifestyle. The dietary habits score was calculated based on weekly intake of vegetables, fruits, red meat, soybean products, and fish: a low-risk lifestyle was defined as eating vegetables and fruits daily; red meat one to six days per week; soybean products four or more days per week; and fish one or more days per week. Each of the above five items scored 1 point, and a score of 4 to 5 was defined as a low-risk lifestyle. The body shape was assessed based on Body Mass Index (BMI) and waist circumference. In this study, participants whose BMI matched the Chinese standard for “normal” and “overweight” (18.5-27.9 kg/m²) and whose waist circumferences also met the normal range of the Chinese standard (<90 cm for men and <85 cm for women) were considered low risk and scored 1 point (26). Then, we added scores for these five areas together. The total score ranges from 0 to 5, and scores positively correlated with lifestyle health.

The questionnaire obtained covariates and included age, sex, and family history of diabetes. Height, weight, waist circumference, and triglycerides were measured at the baseline examination, and BMI was calculated: weight/height² (kg/m²).

### 2.3 Outcome ascertainment

At baseline and follow-up, routine blood tests, fasting glucose, fasting plasma total cholesterol, HDL cholesterol, LDL cholesterol, and triglycerides were measured, relying on the health management centers of each third-class hospital at our cohort sites. Information on the history of T2D and hypoglycemic drugs was collected in a structured questionnaire. According to the Chinese guidelines for preventing and treating type 2 diabetes (2020) (27), participants were defined as having T2D if they met one of the following criteria: 1) those with fasting glucose≥7.0 mmol/L, 2) patients diagnosed with T2D by a hospital, and 3)those taking hypoglycemic drugs.

### 2.4 Statistical analysis

According to the Chinese guidelines for preventing and treating type 2 diabetes (2020), the population was divided into two groups at baseline, whether or not they had the disease (n=5,475). To describe the baseline characteristics of the two groups, we used the Wilcoxon rank sum test and the Kruskal-Wallis *H* test for continuous variables that did not conform to a normal distribution. We used the Pearson chi-square test, or Fisher’s exact test for categorical variables to test for group differences. We adjusted age, sex, family history of diabetes, lifestyle score, and triglyceride levels. We used binary logistic regression to estimate odd ratio (OR) and 95% confidence intervals (CI) between peripheral blood leukocyte and subtypes levels and T2D at baseline cross-sectional. Cox proportional hazard models were used to estimate the risk of incidence of T2D using hazard ratio (HR) and its 95%CI.

We used four logistic regression models and four Cox regression models. Model 1 considered the effects of age and sex on the development of T2D. Model 2 was further adjusted for the effect of body shape based on model 1, a joint assessment of BMI and waist circumference. In Model 3, the body shape variable from the prior model (Model 2) was removed and substituted with a lifestyle score that took into account the five factors of smoking status, alcohol consumption, physical activity, dietary habits, and body shape. Model 3 was also expanded to include the family history of diabetes. Accordingly, model 4 was adjusted by including triglycerides as a continuous variable. Using univariate logistic regression and univariate Cox regression, we investigated the association of different leukocyte subtypes with T2D and selected the leukocyte subtypes with significant results (*P* values < 0.05). Then we constructed ROC curves by R-packet pROC and time-dependent ROC curves by R-packet timeROC (28). All analyses were performed using SPSS 26.0 and R Version 4.1.3. A two-sided *P* value < 0.05 was considered to be of statistical significance.

## 3 Results

### 3.1 Population characteristics

**Table 1** shows the baseline characteristics of participants who underwent cross-sectional analysis. A total of 5,475 participants were included, with a median age of 33.0 years and 28.5% men. Among them, 113(2.06%) were of T2D, and 5,362(97.94%) had no T2D. Individuals with T2D were more likely to be male, older, less educated, divorced, or widowed than those without T2D. In this study, individuals with T2D were more likely to be male and older. These individuals were also more likely to have a history of smoking (current smoker or former smoker), a history of alcohol consumption, to be obese (central obesity, BMI ≥ 28.0 kg/m^2^; either or both), and a family history of diabetes mellitus. Moreover, the leukocyte count and leukocyte subtypes levels in the peripheral blood of these individuals were higher than in the non-diseased population (all *P* ≤ 0.05). Participants excluded from the current study due to missing information are shown in **Supplementary Table 1**.

**Table 1:** Baseline characteristics of participants according to type 2 diabetes.

Characteristics	Total Population	Type 2 diabetes		*P* value
	(n=5475)	Yes(n=113)	No(n=5362)	
Location, n(%)				<0.001
Huaihua	361(6.6)	21(5.8)	340(94.2)	
Changsha	2358(43.1)	44(1.9)	2314(98.1)	
Zhuzhou	1243(22.7)	21(1.7)	1222(98.3)	
Xiangtan	1513(27.6)	27(1.8)	1486(98.2)	
Sex, n(%)				<0.001
male	1560(28.5)	78(5.0)	1482(95.0)	
female	3915(71.5)	35(0.9)	3880(99.1)	
Age, median(IQR)	33.00(11.80)	48.10(11.65)	32.90(11.50)	<0.001
Education level, n(%)				<0.001
High school or below	221(4.0)	21(9.5)	200(90.5)	
college	3825(69.9)	71(1.9)	3754(98.1)	
Graduate or beyond	1429(26.1)	21(1.5)	1408(98.5)	
Marital, n(%)				<0.001
Spinsterhood	1256(22.9)	7(0.6)	1249(99.4)	
Married	4101(74.9)	94(2.3)	4007(97.7)	
Divorced or widowed	114(2.1)	12(10.5)	102(89.5)	
others	4(0.1)	0(0.0)	4(100.0)	
Family history of diabetes mellitus, n(%)				<0.001
Yes	882(16.1)	33(3.7)	849(96.3)	
No	4593(83.9)	80(1.7)	4513(98.3)	
Smoke status, n(%)				<0.001
Current smoker	573(10.5)	33(5.8)	540(94.2)	
Former smoker	65(1.2)	4(6.2)	61(93.8)	
Never smoker	4615(84.3)	71(1.5)	4544(98.5)	
Passive smoking	222(4.1)	5(2.3)	217(97.7)	
Drinking status, n(%)				<0.001
Current drinker	779(14.2)	39(5.0)	740(95.0)	
Former drinker	35(0.6)	3(8.6)	32(91.4)	
Never drinker	4661(85.1)	71(1.5)	4590(98.5)	
Exercise, n(%)				0.006
Yes	2544(46.5)	67(2.6)	2477(97.4)	
No	2931(53.5)	46(1.6)	2885(98.4)	
Body shape, n(%)				<0.001
No.1 shape^a^	4195(76.6)	52(1.2)	4143(98.8)	
No.2 shape^b^	401(7.3)	0(0.0)	401(100.0)	
No.3 shape^c^	421(7.7)	29(6.9)	392(93.1)	
No.4 shape^d^	154(2.8)	2(1.3)	152(98.7)	
No.5 shape^e^	304(5.6)	30(9.9)	274(90.1)	
Diet score, n(%)				0.450
0	297(5.4)	5(1.7)	292(98.3)	
1	1254(22.9)	28(2.2)	1226(97.8)	
2	1693(30.9)	43(2.5)	1650(97.5)	
3	1346(24.6)	21(1.6)	1325(98.4)	
4	767(14.0)	13(1.7)	754(98.3)	
5	118(2.2)	3(2.5)	115(97.5)	
Lifestyle score, n(%)				<0.001
0	71(1.3)	6(8.5)	65(91.5)	
1	286(5.2)	14(4.9)	272(95.1)	
2	970(17.7)	43(4.4)	927(95.6)	
3	2255(41.2)	27(1.2)	2228(98.8)	
4	1567(28.6)	19(1.2)	1548(98.8)	
5	326(6.0)	4(1.2)	322(98.8)	
leukocyte, median(IQR)	6.10(2.08)	6.82(2.12)	6.08(2.06)	<0.001
Lymphocyte, median(IQR)	2.07(0.80)	2.20(0.88)	2.06(0.80)	0.007
Monocyte, median(IQR)	0.34(0.15)	0.38(0.16)	0.33(0.15)	<0.001
Neutrophil, median(IQR)	3.43(1.50)	3.91(1.67)	3.41(1.49)	<0.001
Eosinophil, median(IQR)	0.11(0.11)	0.13(0.12)	0.11(0.10)	0.003
Basophil, median(IQR)	0.02(0.02)	0.03(0.03)	0.02(0.02)	0.001
TC, median(IQR)^f^	4.49(1.12)	5.02(1.23)	4.48(1.11)	<0.001
TG, median(IQR)^g^	0.97(0.82)	2.41(2.60)	0.96(0.79)	<0.001
HDL, median(IQR)^h^	1.46(0.45)	1.12(0.34)	1.46(0.45)	<0.001
LDL, median(IQR)^i^	2.60(0.96)	3.03(1.09)	2.59(0.95)	<0.001

a body shape 1: BMI 18.5-27.9(kg/m^2^), waist circumference < 90 cm of men or < 85 cm of women.

b body shape 2:BMI<18.5(kg/m^2^).

c body shape 3: BMI 18.5-27.9(kg/m^2^), waist circumference ≥ 90 cm of men or ≥ 85 cm of women.

d body shape 4: BMI ≥ 28.0(kg/m^2^), waist circumference < 90 cm of men or < 85 cm of women.

e body shape 5: BMI ≥ 28.0(kg/m^2^), waist circumference ≥ 90 cm of men or ≥ 85 cm of women.

f Total cholesterol.

g Triglycerides.

h High-density lipoprotein.

i Low-density lipoprotein.

### 3.2 Association between peripheral blood leukocyte levels and T2D in a cross-sectional study

We grouped the 5,475 participants at baseline in quintiles for leukocyte count, lymphocyte count, monocyte count, neutrophil count, eosinophil count, and basophil count. The outcome was whether the T2D was present at baseline. We calculated the OR by binary logistic regression. **Table 2** shows that in model 1, after adjusting for sex and age, the risk of prevalence T2D was 2.78 times higher (95% CI: 1.44-5.35) in those with leukocyte count with the highest quintile (Q5) than those with the lowest quintile (Q1). The risk of T2D was 2.42 times higher in people with a lymphocyte count of Q5 than in people with Q1 (95% CI: 1.29-4.55). The OR for T2D was 2.43 (95% CI: 1.30-4.53) for those with a neutrophil count of Q5 compared to those with Q1.

**Table 2:** ORs and 95% CIs for diabetes according to leukocyte and its subsets quartiles at baseline.

Leukocyte (*10^9^/L)	ORs(95%CI)					*P* value
	Q1 (≤4.90)	Q2 (4.91-5.70)	Q3 (5.71-6.47)	Q4 (6.48-7.52)	Q5 (≥7.53)	
DM/nonDM	13/1084	12/1082	23/1077	24/1067	41/1052	
Model 1^a^	1	0.77 (0.34-1.72)	1.56 (0.78-3.16)	1.54 (0.77-3.11)	2.78 (1.44-5.35)	0.001
Model 2^b^	1	0.71 (0.32-1.60)	1.42 (0.70-2.88)	1.16 (0.56-2.37)	2.00 (1.02-3.93)	0.027
Model 3^c^	1	0.76 (0.34-1.71)	1.42 (0.70-2.90)	1.41 (0.69-2.89)	2.43 (1.24-4.75)	0.006
Model 4^d^	1	0.73 (0.32-1.64)	1.22 (0.59-2.52)	1.21 (0.58-2.49)	2.01 (1.02-3.98)	0.035
Lymphocyte (*10^9^/L)	Q1 (≤1.63)	Q2 (1.64-1.93)	Q3 (1.94-2.21)	Q4 (2.22-2.60)	Q5 (≥2.61)	*P* value
DM/nonDM	15/1114	22/1066	21/1054	20/1074	35/1054	
Model 1^a^	1	1.63 (0.83-3.20)	1.68 (0.85-3.34)	1.58 (0.79-3.15)	2.42 (1.29-4.55)	0.096
Model 2^b^	1	1.55 (0.78-3.08)	1.68 (0.84-3.35)	1.31 (0.65-2.64)	2.08 (1.10-3.96)	0.211
Model 3^c^	1	1.47 (0.74-2.90)	1.51 (0.76-3.02)	1.41 (0.70-2.83)	2.10 (1.11-3.97)	0.234
Model 4^d^	1	1.39 (0.70-2.78)	1.38 (0.68-2.78)	1.34 (0.66-2.71)	1.75 (0.91-3.36)	0.573
Monocyte (*10^9^/L)	Q1 (≤0.25)	Q2 (0.26-0.31)	Q3 (0.32-0.37)	Q4 (0.38-0.45)	Q5 (≥0.46)	*P* value
DM/nonDM	14/1123	14/1160	26/1086	24/1046	35/947	
Model 1^a^	1	0.85 (0.40-1.82)	1.53 (0.78-3.00)	1.17 (0.58-2.35)	1.42 (0.72-2.80)	0.399
Model 2^b^	1	0.85 (0.39-1.82)	1.38 (0.70-2.74)	0.91 (0.45-1.87)	0.98 (0.49-1.98)	0.576
Model 3^c^	1	0.84 (0.39-1.80)	1.52 (0.77-3.00)	1.00 (0.49-2.04)	1.23 (0.61-2.46)	0.417
Model 4^d^	1	0.89 (0.41-1.93)	1.47 (0.73-2.96)	0.96 (0.46-1.99)	1.29 (0.64-2.61)	0.490
Neutrophil (*10^9^/L)	Q1 (≤2.60)	Q2 (2.61-3.17)	Q3 (3.18-3.72)	Q4 (3.73-4.48)	Q5 (≥4.49)	*P* value
DM/nonDM	15/1098	13/1078	24/1079	21/1066	40/1041	
Model 1^a^	1	0.68 (0.32-1.45)	1.39 (0.71-2.71)	1.18 (0.59-2.35)	2.43 (1.30-4.53)	0.001
Model 2^b^	1	0.66 (0.30-1.41)	1.19 (0.60-2.34)	0.90 (0.44-1.81)	1.77 (0.93-3.36)	0.024
Model 3^c^	1	0.63 (0.29-1.36)	1.38 (0.70-2.71)	1.04 (0.52-2.10)	2.16 (1.14-4.08)	0.002
Model 4^d^	1	0.56 (0.26-1.23)	1.15 (0.56-2.29)	0.81 (0.39-1.67)	1.80 (0.95-3.44)	0.005
Eosinophil (*10^9^/L)	Q1 (≤0.06)	Q2 (0.07-0.09)	Q3 (0.10-0.13)	Q4 (0.14-0.20)	Q5 (≥0.21)	*P* value
DM/nonDM	17/1325	19/1004	23/1068	24/989	30/976	
Model 1^a^	1	1.23 (0.63-2.41)	1.32 (0.69-2.53)	1.19 (0.62-2.28)	1.25 (0.66-2.34)	0.942
Model 2^b^	1	1.13 (0.57-2.25)	1.13 (0.58-2.18)	0.99 (0.51-1.91)	1.08 (0.57-2.05)	0.988
Model 3^c^	1	1.10 (0.55-2.19)	1.22 (0.63-2.35)	1.05 (0.54-2.03)	1.14 (0.60-2.17)	0.978
Model 4^d^	1	1.06 (0.53-2.12)	1.08 (0.55-2.10)	0.88 (0.45-1.74)	1.04 (0.55-1.99)	0.973
Basophil (*10^9^/L)	Q1 (≤0.010)	Q2 (0.011-0.020)	Q3 (0.021-0.030)	Q4 (0.031-0.040)	Q5 (≥0.041)	*P* value
DM/nonDM	31/1856	22/1325	22/1045	14/576	24/560	
Model 1^a^	1	0.83 (0.47-1.45)	0.89 (0.51-1.58)	0.95 (0.49-1.84)	1.52 (0.86-2.67)	0.323
Model 2^b^	1	0.78 (0.44-1.38)	0.83 (0.46-1.48)	0.88 (0.45-1.72)	1.26 (0.71-2.25)	0.573
Model 3^c^	1	0.88 (0.50-1.56)	0.97 (0.55-1.73)	0.95 (0.49-1.86)	1.54 (0.86-2.73)	0.422
Model 4^d^	1	0.98 (0.55-1.76)	1.10 (0.61-1.98)	1.02 (0.51-2.01)	1.42 (0.78-2.59)	0.777

Q, quintile.

a Model 1 adjusted for age and sex.

b Model 2 adjusted for age, sex, and body shape.

c Model 3 adjusted for age, sex, lifestyle score, and family history of diabetes.

d Model 4 adjusted for age, sex, lifestyle score, family history of diabetes, and triglycerides. * multiplication sign.

Model 2 further adjusted for body shape: people with a leukocyte count of Q5 had 2.00 times (95% CI: 1.02-3.93) the risk of developing T2D than those with a leukocyte count of Q1. People with a lymphocyte count of Q5 had a 2.08 times higher risk of developing T2D than those with a lymphocyte count of Q1 (95% CI: 1.10-3.96).

Model 3 adjusted for a lifestyle score and added a family history of diabetes. The results showed that people with a leukocyte count at Q5 had a 2.43 times higher risk of developing T2D than those with a leukocyte at Q1 (95% CI: 1.24-4.75). The OR for T2D was 2.10 (95% CI: 1.11-3.97) for those with a lymphocyte count of Q5 compared to those with Q1. The OR for T2D was 2.16 (95% CI: 1.14-4.08) for those with a neutrophil count of Q5 compared to those with Q1.

Model 4 was adjusted by adding triglycerides as a continuous type variable to the model. The results showed that the OR for T2D was 2.01 (95% CI: 1.02-3.98) for those with Q5 compared to those with Q1 leukocyte count. Furthermore, the leukocyte count and neutrophils had a total *P* value < 0.05 in all four models.

**Figure 2:**
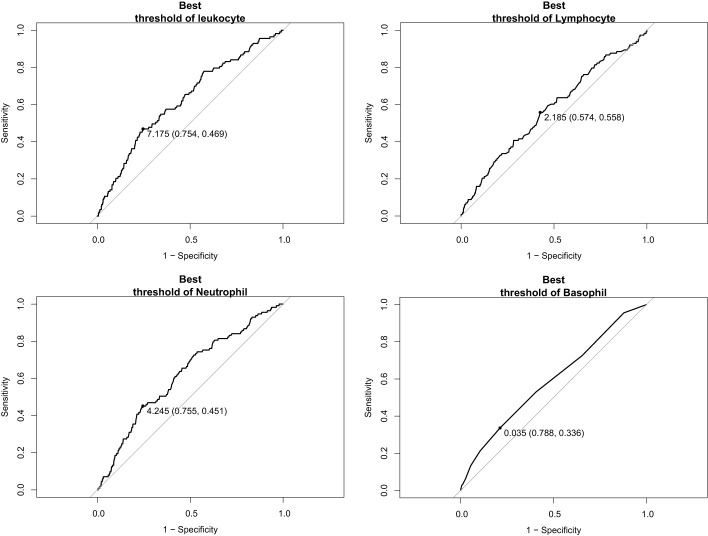
ROC curves and best thresholds for leukocyte, lymphocyte, neutrophil, and basophil.

**Figure 3:**
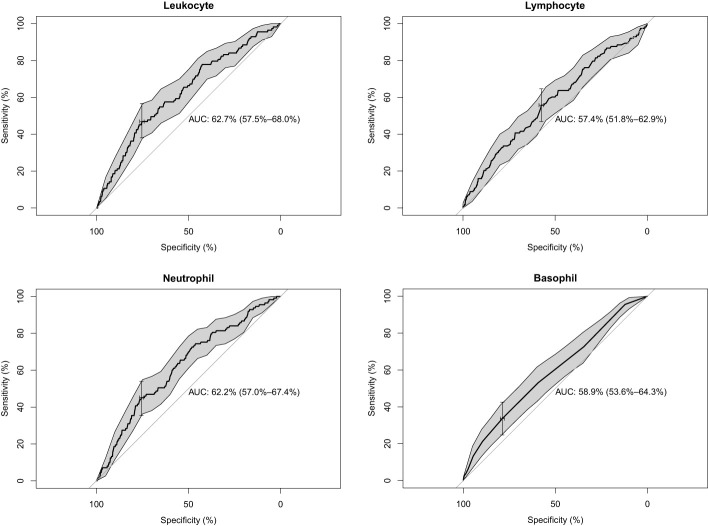
AUCs and confidence intervals of leukocyte, lymphocyte, neutrophil, and basophil.

We included the leukocyte count, lymphocyte count, monocyte count, neutrophil count, and basophil count of these 5,475 participants as quantitative variables in a logistic regression model. A univariate logistic regression analysis of the relationship between subtypes of leukocytes and T2D was then performed. The results showed that the results for leukocyte count, lymphocyte count, neutrophil count, and basophil count were significant (*P*-value < 0.05) (**Table 3**). Using ROC curves, we estimated the predictive diagnosis of T2D based on baseline leukocyte count, lymphocyte count, monocyte count, neutrophil count, and basophil count. We calculated the best thresholds for these four indicators (**Figure 2**). The corresponding AUCs are shown in **Figure 3**: the above four indicators have some predictive value for T2D with AUCs > 0.5, [AUC: (leukocyte count 0.63 > neutrophil count 0.62 > basophil count 0.59 > lymphocyte count 0.57)].

**Table 3:** Univariate regression analysis.

Subtype of lekocytes	DM/nonDM 113/5362			DM/nonDM 29/4510		
	OR	95%OR	P-value	HR	95%HR	*P*-value
Leukocyte	1.27	(1.15-1.40)	<0.001	1.27	(1.04-1.54)	0.017
Lymphocyte	1.53	(1.16-2.03)	0.003	1.29	(0.73-2.26)	0.385
Neutrophil	1.29	(1.14-1.45)	<0.001	1.31	(1.03-1.67)	0.027
Monocyte	0.93	(0.65-1.34)	0.698	39.80	(3.44-460.34)	0.003
Eosinophil	2.85	(0.69-11.82)	0.149	11.41	(1.46-89.27)	0.02
Basophil	1.73E+8	(2.13E+4-1.40E+12)	<0.001	20.67	(2.61E-8-1.64E+10)	0.772

### 3.3 Associations of different peripheral blood leukocytes exposure levels with incidence of T2D

Excluding the 113 individuals with the disease at baseline, the remaining 5,362 individuals were subjected to longitudinal analysis. **Table 4** shows the baseline characteristics of participants who underwent longitudinal analysis with a median age of 32.9 years and 27.6% males. In this study, those in Q5 were likelier to be male and had a lower lifestyle score than those in Q1 of leukocyte count.

**Table 4:** Baseline characteristics of people without type 2 diabetes at baseline across leukocyte quintiles.

Characteristics	Total	Leukocyte(*10^9^/L)					*P* value
	(n=5362)	Q1 (≤4.89)	Q2 (4.90-5.69)	Q3 (5.70-6.46)	Q4 (6.57-7.50)	Q5 (≥7.51)	
Location, n(%)							0.003
Huaihua	340(6.3)	67 (19.7)	62 (18.2)	78 (22.9)	74 (21.8)	59 (17.4)	
Changsha	2314 (43.2)	496 (21.4)	490 (21.2)	482 (20.8)	435 (18.8)	411 (17.8)	
Zhuzhou	1222 (22.8)	223 (18.2)	240 (19.6)	242 (19.8)	237 (19.4)	280 (22.9)	
Xiangtan	1486 (27.7)	288 (19.4)	286 (19.2)	273 (18.4)	322 (21.7)	317 (21.3)	
Sex, n(%)							<0.001
male	1482 (27.6)	186 (12.6)	260 (17.5)	305 (20.6)	342 (23.1)	389 (26.2)	
female	3880 (72.4)	888 (22.9)	818 (21.1)	770 (19.8)	726 (18.7)	678 (17.5)	
Age, median(IQR)	32.90 (11.50)	34.00 (12.00)	33.00 (12.02)	33.00 (11.50)	32.15 (11.27)	32.00 (10.20)	<0.001
Education level, n(%)							0.902
High school or below	200 (3.7)	42 (21.0)	39 (19.5)	39 (19.5)	33 (16.5)	47 (23.5)	
college	3754 (70.0)	751 (20.0)	750 (20.0)	760 (20.2)	758 (20.2)	735 (19.6)	
Graduate or beyond	1408 (26.3)	281 (20.0)	289 (20.5)	276 (19.6)	277 (19.7)	285 (20.2)	
Marital, n(%)							0.246
Spinsterhood	1249 (23.3)	222 (17.8)	252 (20.2)	239 (19.1)	277 (22.2)	259 (20.7)	
Married	4007 (74.7)	826 (20.6)	805 (20.1)	812 (20.3)	772 (19.3)	792 (19.8)	
Divorced or widowed	102 (1.9)	25 (24.5)	19 (18.6)	23 (22.5)	19 (18.6)	16 (15.7)	
others	4 (0.1)	1 (25.0)	2 (50.0)	1 (25.0)	0 (0.0)	0 (0.0)	
Family history of diabetes mellitus, n(%)							0.619
Yes	849 (15.8)	157 (18.5)	166 (19.6)	169 (19.9)	180 (21.2)	177 (20.8)	
No	4513 (84.2)	917 (20.3)	912 (20.2)	906 (20.1)	888 (19.7)	890 (19.7)	
Smoke status, n(%)							<0.001
Current smoker	540 (10.1)	32 (5.9)	68 (12.6)	106 (19.6)	134 (24.8)	200 (37.0)	
Former smoker	61 (1.1)	5 (8.2)	11 (18.0)	17 (27.9)	13 (21.3)	15 (24.6)	
Never smoker	4544 (84.7)	995 (21.9)	957 (21.1)	907 (20.0)	876 (19.3)	809 (17.8)	
Passive smoking	217 (4.0)	42 (19.4)	42 (19.4)	45 (20.7)	45 (20.7)	43 (19.8)	
Drinking status, n(%)							<0.001
Current drinker	740 (13.8)	101 (13.6)	142 (19.2)	150 (20.3)	172 (23.2)	175 (23.6)	
Former drinker	32 (0.6)	2 (6.3)	5 (15.6)	9 (28.1)	8 (25.0)	8 (25.0)	
Never drinker	4590 (85.6)	971 (21.2)	931 (20.3)	916 (20.0)	888 (19.3)	884 (19.3)	
Exercise, n(%)							0.004
Yes	2477 (46.2)	516 (20.8)	536 (21.6)	483 (19.5)	494 (19.9)	448 (18.1)	
No	2885 (53.8)	558 (19.3)	542 (18.8)	592 (20.5)	574 (19.9)	619 (21.5)	
Body shape, n(%)							<0.001
No.1 shape^a^	4142 (77.2)	892 (21.5)	875 (21.1)	833 (20.1)	797 (19.2)	745 (18.0)	
No.2 shape^b^	405 (7.6)	109 (26.9)	100 (24.7)	85 (21.0)	64 (15.8)	47 (11.6)	
No.3 shape^c^	389 (7.3)	37 (9.5)	56 (14.4)	85 (21.9)	98 (25.2)	113 (29.0)	
No.4 shape^d^	152 (2.8)	23 (15.1)	23 (15.1)	33 (21.7)	34 (22.4)	39 (25.7)	
No.5 shape^e^	274 (5.1)	13 (4.7)	24 (8.8)	39 (14.2)	75 (27.4)	123 (54.5)	
Diet score, n(%)							0.264
0	292 (5.4)	50 (17.1)	57 (19.5)	70 (24.0)	62 (21.2)	53 (18.2)	
1	1226 (22.9)	220 (17.9)	251 (20.5)	246 (20.1)	257 (21.0)	252 (20.6)	
2	1650 (30.8)	334 (20.2)	326 (19.8)	344 (20.8)	325 (19.7)	321 (19.5)	
3	1325 (24.7)	296 (22.3)	270 (20.4)	243 (18.3)	251 (18.9)	265 (20.0)	
4	754 (14.1)	159 (21.1)	146 (19.4)	143 (19.0)	156 (20.7)	150 (19.9)	
5	115 (2.1)	15 (13.0)	28 (24.3)	29 (25.2)	17 (14.8)	26 (22.6)	
Lifestyle score, n(%)							<0.001
0	65 (1.2)	0 (0.0)	6 (9.2)	16 (24.6)	16 (24.6)	27 (41.5)	
1	271 (5.1)	19 (7.0)	46 (17.0)	53 (19.6)	66 (24.4)	87 (32.1)	
2	925 (17.3)	157 (17.0)	144 (15.6)	194 (21.0)	207 (22.4)	223 (24.1)	
3	2226 (41.5)	449 (20.2)	465 (20.9)	441 (19.8)	431 (19.4)	440 (19.8)	
4	1552 (28.9)	377 (24.3)	341 (22.0)	308 (19.8)	285 (18.4)	241 (15.5)	
5	323 (6.0)	72 (22.3)	76 (23.5)	63 (19.5)	63 (19.5)	49 (15.2)	
Lymphocyte, median(IQR)	2.06 (0.80)	1.61 (0.44)	1.90 (0.52)	2.10 (0.57)	2.31 (0.68)	2.63 (0.90)	<0.001
Monocyte, median(IQR)	0.33 (0.15)	0.25 (0.08)	0.30 (0.10)	0.34 (0.11)	0.38 (0.12)	0.46 (0.15)	<0.001
Neutrophil, median(IQR)	3.41 (1.49)	2.32 (0.62)	2.94 (0.59)	3.46 (0.61)	4.00 (0.76)	5.14 (1.36)	<0.001
Eosinophil, median(IQR)	0.11 (0.10)	0.07 (0.07)	0.10 (0.08)	0.11 (0.11)	0.12 (0.12)	0.15 (0.14)	<0.001
Basophil, median(IQR)	0.02 (0.02)	0.02 (0.01)	0.02 (0.02)	0.02 (0.02)	0.02 (0.03)	0.03 (0.03)	<0.001
TC, median(IQR)^f^	4.48 (1.11)	4.46 (1.10)	4.39 (1.14)	4.49 (1.06)	4.51 (1.12)	4.55 (1.11)	0.002
TG, median(IQR)^g^	0.96 (0.79)	0.79 (0.52)	0.87 (0.53)	0.97 (0.77)	1.06 (0.93)	1.23 (1.11)	<0.001
HDL, median(IQR)^h^	1.46 (0.45)	1.58 (0.41)	1.51 (0.42)	1.45 (0.44)	1.40 (0.40)	1.33 (0.45)	<0.001
LDL, median(IQR)^i^	2.59 (0.95)	2.57 (0.93)	2.49 (0.96)	2.58 (0.96)	2.65 (1.00)	2.68 (0.95)	<0.001

Q, quintile.

a body shape 1: BMI 18.5-27.9(kg/m^2^), waist circumference < 90 cm of men or < 85 cm of women.

b body shape 2:BMI<18.5(kg/m^2^).

c body shape 3: BMI 18.5-27.9(kg/m^2^), waist circumference ≥ 90 cm of men or ≥ 85 cm of women.

d body shape 4: BMI ≥ 28.0(kg/m^2^), waist circumference < 90 cm of men or < 85 cm of women.

e body shape 5: BMI ≥ 28.0(kg/m^2^), waist circumference ≥ 90 cm of men or ≥ 85 cm of women.

f Total cholesterol.

g Triglycerides.

h High-density lipoprotein.

i Low-density lipoprotein.

The average follow-up time was 12 months, and 5,013 people were followed up, with a missing rate of 6.51% (349/5362). Of these, the incidence proportion of T2D was 0.58% (29/5013), and information on follow-up outcomes was missing for 355 individuals. Baseline information for these 5,013 individuals is described in detail in **Supplementary Table 2**. The results suggested that those with a new onset were likelier to have high levels of peripheral blood leukocyte and its subtypes at baseline.

We further grouped these 5,013 participants in quintiles of leukocyte and its subtypes at baseline, calculated the follow-up time for each individual, and used the presence or absence of T2D at follow-up as an outcome indicator. Using the Cox proportional hazard model, the hazard ratio (HR) of developing T2D was calculated for those who did not have T2D at baseline. The models were adjusted for variables consistent with the logistic regression described above.

The results showed that in model 1, people with a leukocyte count of Q5 had up to 10 times more risk of disease compared to those with a leukocyte count of Q1 (HR: 10.09, 95% CI: 1.30-78.23). The HR for T2D was 5.73 (95% CI: 1.19-27.72) in people with a neutrophil count of Q5 compared to those with a neutrophil count of Q1.

In model 3, people with a leukocyte count of Q5 had a risk of the disease up to 8.32 times higher (HR: 8.32, 95% CI: 1.05-65.73) compared to people with a leukocyte count of Q1. People with a neutrophil count of Q5 had up to 4 times more risk of disease than those with a neutrophil count of Q1 (HR: 4.95, 95% CI: 1.00-24.45).

In model 4, those with a leukocyte count of Q5 had a more than 8-fold increased risk of disease compared to those with a leukocyte count of Q1 (HR: 8.43, 95% CI: 1.06-66.92). Those with a neutrophil count of Q5 had a more than 5-fold increased risk of disease compared to those with a neutrophil count of Q1 (HR: 5.05, 95% CI: 1.01-25.29) (**Table 5**).

**Table 5:** HR for developing diabetes in nondiabetes patients across leukocyte and its subsets quintiles.

Leukocyte (*10^9^/L)	HR(95%CI)					*P* value
	Q1 (≤4.87)	Q2 (4.88-5.67)	Q3 (5.68-6.44)	Q4 (6.45-7.46)	Q5 (≥7.47)	
DM/nonDM	1/943	8/930	3/943	4/912	13/901	
Model 1^a^	1	6.14 (0.76-49.66)	2.63 (0.27-25.41)	2.72 (0.30-24.62)	10.09 (1.30-78.23)	0.026
Model 2^b^	1	5.09 (0.63-41.25)	2.17 (0.22-21.10)	1.73 (0.19-15.98)	5.77 (0.72-46.18)	0.100
Model 3^c^	1	5.00 (0.61-41.19)	2.22 (0.23-21.74)	2.37 (0.26-21.67)	8.32 (1.05-65.73)	0.047
Model 4^d^	1	5.00 (0.61-41.19)	2.22 (0.23-21.79)	2.39 (0.26-21.87)	8.43 (1.06-66.92)	0.047
Lymphocyte (*10^9^/L)	Q1 (≤1.62)	Q2 (1.63-1.91)	Q3 (1.92-2.20)	Q4 (2.21-2.60)	Q5 (≥2.61)	*P* value
DM/nonDM	5/966	8/908	4/943	4/945	8/867	
Model 1^a^	1	1.65 (0.54-5.06)	0.87 (0.23-3.26)	0.71 (0.18-2.72)	1.45 (0.47-4.52)	0.607
Model 2^b^	1	1.17 (0.37-3.70)	0.90 (0.24-3.35)	0.61 (0.16-2.30)	1.18 (0.37-3.76)	0.837
Model 3^c^	1	1.40 (0.45-4.39)	0.79 (0.21-2.97)	0.59 (0.15-2.32)	1.30 (0.41-4.10)	0.643
Model 4^d^	1	1.40 (0.44-4.41)	0.79 (0.21-2.97)	0.59 (0.15-2.32)	1.30 (0.41-4.14)	0.647
Monocyte (*10^9^/L)	Q1 (≤0.25)	Q2 (0.26-0.31)	Q3 (0.32-0.36)	Q4 (0.37-0.44)	Q5 (≥0.45)	*P* value
DM/nonDM	2/1031	5/1033	3/814	8/946	11/805	
Model 1^a^	1	3.65 (0.64-20.72)	2.50 (0.38-16.65)	3.76 (0.72-19.69)	4.19 (0.84-21.03)	0.488
Model 2^b^	1	2.63 (0.48-14.28)	1.78 (0.28-11.34)	2.15 (0.43-10.82)	2.08 (0.42-10.37)	0.856
Model 3^c^	1	3.98 (0.67-23.53)	2.39 (0.35-16.38)	3.93 (0.72-21.52)	3.94 (0.74-20.94)	0.515
Model 4^d^	1	3.98 (0.67-23.52)	2.38 (0.35-16.33)	3.92 (0.72-21.46)	3.92 (0.73-20.90)	0.517
Neutrophil (*10^9^/L)	Q1 (≤2.59)	Q2 (2.60-3.15)	Q3 (3.16-3.69)	Q4 (3.70-4.44)	Q5 (≥4.45)	*P* value
DM/nonDM	2/948	5/925	5/923	6/923	11/910	
Model 1^a^	1	3.06 (0.54-17.23)	2.54 (0.45-14.34)	3.22 (0.59-17.57)	5.73 (1.19-27.72)	0.182
Model 2^b^	1	2.25 (0.42-12.15)	1.53 (0.28-8.445)	1.78 (0.33-9.54)	3.01 (0.62-14.68)	0.561
Model 3^c^	1	2.96 (0.52-16.87)	2.22 (0.39-12.64)	3.06 (0.55-16.97)	4.95 (1.00-24.45)	0.270
Model 4^d^	1	2.99 (0.52-17.10)	2.26 (0.39-12.99)	3.11 (0.55-17.45)	5.05 (1.01-25.29)	0.269
Eosinophil (*10^9^/L)	Q1 (≤0.06)	Q2 (0.07-0.09)	Q3 (0.10-0.13)	Q4 (0.14-0.19)	Q5 (≥0.20)	*P* value
DM/nonDM	4/1184	2/885	3/921	7/748	13/891	
Model 1^a^	1	0.45 (0.08-2.49)	0.52 (0.11-2.56)	1.62 (0.47-5.57)	2.08 (0.67-6.48)	0.123
Model 2^b^	1	0.31 (0.05-1.85)	0.47 (0.10-2.20)	1.17 (0.34-4.09)	1.47 (0.46-4.66)	0.228
Model 3^c^	1	0.39 (0.07-2.20)	0.44 (0.09-2.21)	1.57 (0.45-5.48)	1.86 (0.58-5.93)	0.123
Model 4^d^	1	0.39 (0.07-2.20)	0.44 (0.09-2.21)	1.56 (0.45-5.48)	1.86 (0.58-5.93)	0.124
Basophil (*10^9^/L)	Q1 (≤0.010)	Q2 (0.011-0.020)	Q3 (0.021-0.030)	Q4 (0.031-0.040)	Q5 (≥0.041)	*P* value
DM/nonDM	8/1574	5/1187	7/921	4/483	5/464	
Model 1^a^	1	0.42 (0.13-1.37)	0.79 (0.28-2.25)	0.70 (0.20-2.43)	0.72 (0.23-2.28)	0.706
Model 2^b^	1	0.44 (0.14-1.38)	0.61 (0.21-1.82)	0.64 (0.19-2.19)	0.62 (0.20-1.94)	0.712
Model 3^c^	1	0.41 (0.13-1.35)	0.78 (0.27-2.25)	0.70 (0.20-2.40)	0.75 (0.24-2.39)	0.694
Model 4^d^	1	0.41 (0.13-1.35)	0.78 (0.27-2.25)	0.69 (0.20-2.40)	0.75 (0.23-2.39)	0.696

Q, quintile.

a Model 1 adjusted for age and sex.

b Model 2 adjusted for age, sex, and body shape.

c Model 3 adjusted for age, sex, lifestyle score, and family history of diabetes.

d Model 4 adjusted for age, sex, lifestyle score, family history of diabetes, and triglycerides. * multiplication sign.

We included the leukocyte count, lymphocyte count, monocyte count, neutrophil count, and basophil count of these 5013 participants as quantitative variables in the Cox regression model. Using univariate Cox regression, we investigated the association between different leukocyte subtypes and T2D. We selected the leukocyte subtypes with significant results (*P* values < 0.05): leukocyte count, neutrophil count, monocyte count, and basophil count (**Table 3**). Time-dependent ROC curves were plotted according to different follow-up times to estimate the predictive diagnosis of T2D by the four indicators mentioned above. The analysis revealed that the follow-up time of these 4658 (5013-355) participants ranged from 4 months to 36 months, with a mean follow-up time of 12 months, with new cases occurring in the follow-up population from 10 months to 36 months. The corresponding AUCs are shown in **Table 6**: the above four indicators had some predictive value for T2D, except for 10 and 36 months, where the time points of AUCs were >0.5. Notably, in predicting the occurrence of T2D at follow-up, the AUC for eosinophil count was the same as the leukocyte count at month 23 (AUC=0.60); the AUC for eosinophil count was higher than the AUC for the other four metrics at all other time points. The specific time-dependent ROC curves are shown in the **Supplementary File** (**Supplementary Figure 1**).

**Table 6:** Best threshold, areas under the time-dependent receiver operating characteristic curves for leukocyte, neutrophil, monocyte, and eosinophil predicting future diabetes risk.

Time (months)	DM/nonDM	Leukocyte			Neutrophil			Monocyte			Eosinophil		
		Best threshold	UC	AUC,95%CI	Best threshold	AUC	AUC,95%CI	Best threshold	AUC	AUC,95%CI	Best threshold	AUC	AUC,95%CI
10	5/530	7.53	NA	NA	4.65	NA	NA	0.4	NA	NA	0.27	NA	NA
11	4/1739	7.53	0.59	(0.24-0.95)	4.25	0.59	(0.26-0.93)	0.48	0.57	(0.25-0.88)	0.16	0.65	(0.33-0.98)
12	6/1240	7.53	0.65	(0.41-0.88)	3.78	0.64	(0.41-0.86)	0.48	0.58	(0.38-0.79)	0.16	0.70	(0.52-0.89)
13	3/459	7.53	0.59	(0.43-0.76)	3.78	0.60	(0.46-0.75)	0.33	0.55	(0.37-0.72)	0.15	0.69	(0.55-0.83)
14	4/312	7.27	0.57	(0.43-0.71)	2.56	0.62	(0.49-0.74)	0.43	0.63	(0.48-0.78)	0.11	0.59	(0.41-0.77)
23	2/29	6.84	0.60	(0.43-0.76)	4.41	0.53	(0.35-0.70)	0.41	0.68	(0.52-0.84)	0.14	0.60	(0.47-0.74)
25	2/41	5.53	0.65	(0.49-0.80)	3.51	0.64	(0.48-0.80)	0.36	0.60	(0.40-0.81)	0.19	0.73	(0.58-0.87
27	2/9	5.45	0.68	(0.39-0.96)	3.18	0.67	(0.36-0.97)	0.29	0.63	(0.32-0.94)	0.16	0.68	(0.33-1.02)
36	1/2	5.21	NA	NA	2.54	NA	NA	0.29	NA	NA	0.05	NA	NA

**Figure 4:**
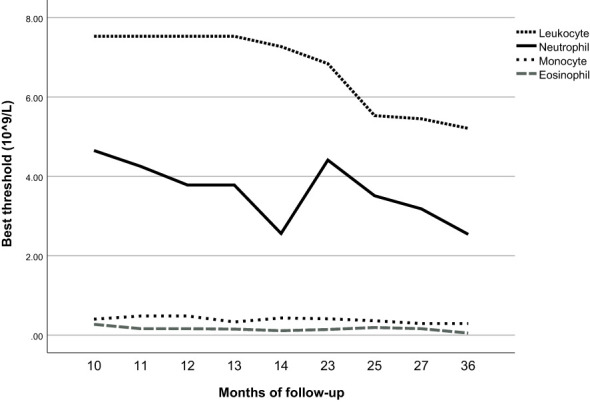
Best thresholds for timeROC of leukocyte, neutrophil, eosinophil, and basophil.

Based on the time-dependent ROC curves, we also calculated the best thresholds for the above four indicators (**Figure 4**). We found that the best thresholds for leukocyte count and neutrophil count fluctuated widely (range of best thresholds for leukocyte count: 5.21-7.53 and neutrophil count: 2.54-4.65). In contrast, the best thresholds for monocyte count and eosinophil count were more stable (range of best thresholds for monocyte count: 0.57-0.67 and eosinophil count: 0.59-0.73). The 355 participants excluded as censored items from the Cox proportion hazard model due to missing information on follow-up outcomes are shown in the **Supplementary File** (**Supplementary Table 3**).

## 4 Discussion

In this longitudinal study, the prevalence of T2D was 2.06% at baseline, and the cumulative incidence density of T2D was 4.0/1000 person-years after a mean follow-up of 12 months. Cross-sectional results showed that sex, age, unhealthy lifestyle, and body shape were associated with T2D and that all patients had higher levels of leukocytes in peripheral blood than those in the non-diabetic population. After adjusting for the effects of these covariates, individuals with a leukocyte count in the highest quintile (Q5) had more than twice the risk of incidence of T2D than those with a leukocyte count in the lowest quintile (Q1). The results of the follow-up study corroborated that people in the highest quintile of leukocyte count and neutrophil count had more than five times the risk of incident T2D than others. Notably, the higher the quintile of leukocyte count, the more likely these people were to have smoking, alcohol consumption, lack of physical activity, and obesity. Overall, the higher the level of the leukocyte count quintile, the more likely these individuals were to have a lower lifestyle score (11). In summary, an increase in leukocytes and subsets was associated with an increased risk of incident T2D, which is generally consistent with previous studies (18, 19).

Peripheral blood is the blood in the body other than the bone marrow. Peripheral blood is mainly composed of 3 types of cells, namely leukocytes, red blood cells, and platelets, referred to as peripheral blood cells. The five peripheral blood leukocytes are neutrophils, basophils, eosinophils, monocytes, and lymphocytes. Neutrophils are the most numerous white blood cells with chemotactic and phagocytic functions. Basophils can initiate an inflammatory response, which has the same function as the secretions of mast cells. Eosinophils are lysosomes with many eosinophilic granules in their cytoplasm. Monocytes are the largest leukocytes in volume and can further differentiate into cells with phagocytic functions. Lymphocytes can be divided into thymus-dependent lymphocytes, bone marrow-dependent lymphocytes, and natural killer cells (29).

Available research evidence suggests that T2D is a chronic disease characterized by hyperglycemia, pancreatic β-cell dysfunction, and insulin resistance that is associated with cellular stress responses, including endoplasmic reticulum stress, lipotoxicity, and glucotoxicity (30). Instead, all these cellular stress responses induce a chronic low-grade inflammatory state and an innate immune response (31). Animal evidence suggests that neutrophils secrete a serine protease that causes insulin resistance. Deleting this protease reduces adipose tissue inflammation and improves glucose tolerance and insulin sensitivity (32). The research found that lymphocytes play an important role in inflammation associated with obesity. Two lymphocyte populations, B cells and T cells, are the source of inflammation associated with obesity and/or T2D (33, 34). Pro-inflammatory lymphocytes can mediate macrophage polarization in obese patients with adipose tissue (35). One reason for this is due to increased expression of chemokine CXCR3 on T cells, which causes an increase in adipose tissue lymphocytes due to the recruitment of T cells in adipose tissue (36). The results of the cellular experiments suggest that T2D may also be associated with an autoimmune component: fat individuals have a small diversity of T-cell receptor pools (TCRs), and the limited TCRs are in turn combined with increased autoimmune antibodies in B cells of insulin-resistant subjects (37).

The results of our study largely support these points. According to our cross-sectional study, the results of univariate logistic regression of both neutrophils and lymphocytes with T2D were significant (*P*-value < 0.05). After further adjustment for other covariates, the relationship between lymphocytes and T2D was significant in logistic regression model 1, model 2, and model 3. People with a lymphocyte count of Q5 have more than twice the risk of developing T2D than those with Q1. The relationship between neutrophils and T2D has significance in model 1 and model 3. People with a neutrophil count of Q5 have more than twice the risk of developing T2D as people with Q1. The ROC curve results also indicate that neutrophils and lymphocytes have some predictive values for T2D. The results of the longitudinal analysis showed that the results of the univariate Cox regression of the relationship between neutrophils and T2D were significant (*P*-value < 0.05). After adjusting for other covariates, the relationship between neutrophils and T2D was significant in model 3 and model 4. People with a neutrophil count of Q5 had more than four times the HR for T2D compared to those with Q1. The results of the time-dependent ROC curve also showed its predictive value for T2D. However, we have not yet found positive results for lymphocytes and the risk of developing T2D in a longitudinal analysis. It may be related to the fact that there is currently only one follow-up data from this study. Therefore, we will increase the follow-up time in future studies and further investigate the association between leukocyte subtypes and the risk of developing T2D.

Regarding the mechanisms of leukocyte changes in T2D patients, previous studies have suggested that this may be due to cellular stress. This cellular molecular process has been described in detail in reference 10 (10). Oxidative stress and lipotoxicity are linked to T2D, which can further lead to insulin resistance, islet dysfunction, and inflammatory reactions (3). Previous research has shown that T2D, an inflammatory disease, is characterized by inflammation in the form of a more significant number of leukocytes in the peripheral blood of patients with T2D compared to patients with type 1 diabetes and healthy individuals (18). It has also been proposed that the mechanism of leukocyte changes in the body of T2D patients may be related to obesity. Obesity may raise the risk of other chronic diseases incidence (38–41). Triglycerides are the main component of lipids and are stored in adipose tissue (42). Adipose tissue has a variety of functions (8, 43, 44), and obesity causes adipocyte hypertrophy, thereby increasing insulin resistance and promoting inflammation (42, 45, 46). This claim was corroborated in another prospective cohort study (47): Leukocyte counts can predict the risk of developing T2D in obese people. Similarly, our findings support the differential nature of leukocyte counts in T2D patients versus those without the disease. Furthermore, our study considered the effect of dietary habits on T2D and controlled for it as a covariate. Instead of including BMI and waist circumference as two separate variables in the model, we combined them into a new variable, “body shape,” and considered them together.

T2D may spread to other body systems as a chronic disease if effective preventive and intervention measures are not. It causes complications, including cardiovascular disease (48, 49), diabetic nephropathy (50–52), and diabetic retinopathy (53). It creates a serious disease burden that can affect the patient’s quality of life and life expectancy. A cross-sectional study showed that The prevalence of metabolic syndrome in the Korean population was positively correlated with peripheral blood levels of leukocyte count, eosinophils, monocyte count, and basophils (54). However, in our cross-sectional study, only differences in leukocyte count and subsets levels were found between the two groups of people with and without T2D. A positive correlation between T2D and the three subsets mentioned above could not yet be indicated. Another cross-sectional study in Chinese adults showed that eosinophil levels in peripheral blood were negatively associated with the risk of T2D (55). The following are some potential causes for this occurrence: 1) Changes in inflammatory factor levels in the organism during the one-year follow-up may not only be related to changes in T2D but also influenced by colds or other diseases. 2) Participants in this study came from Hunan Province in China. While the sample size was massive, the study’s results may not be generalizable to other regions of China.

Current studies on the relationship between the risk of T2D and environmental-lifestyle factors suggest that energy intake and metabolic imbalance are the leading causes of the increased incidence of T2D (4, 56). These are generally consistent with our findings: People with T2D and those with the incidence of the disease are more likely to have high peripheral blood leukocyte levels and low lifestyle scores. Another research proposed that the relationship between alcohol and the incidence of T2D was primarily related to alcohol intake: moderate intake was negatively associated with incidence, and the association between heavy alcohol intake and incidence was unclear (57). Furthermore, in the results of this study, it was found that among those with normal blood glucose at baseline, those with total peripheral blood leukocyte counts in the highest quintile were more likely to have a history of smoking and alcohol consumption. The primary strategy for reducing the risk of T2D remains weight reduction, i.e., eating and moving in balance, as suggested by the pilot study on lifestyle changes to prevent diabetes (56). That can effectively reduce the incidence of diabetes. Our findings are generally consistent with this view.

The strength of this study is the large sample size obtained from an established representative cohort of Chinese public employees. In addition, we assessed the association between peripheral blood leukocyte levels and T2D by controlling for general demographic characteristics, lifestyles, and other covariates. However, there are still some potential limitations of the present study. Firstly, the information on lifestyle was mainly self-reported, which allowed us to assess only over a certain period, so information bias was inevitable. Secondly, participants in this study came from Hunan Province in China. While the sample size was massive, the study’s results may not be generalizable to other regions of China. Future multi-province or multi-country studies are needed to validate our findings further. Thirdly, plasma leukocyte levels may be affected by other health conditions (such as colds or other diseases that have not been measured or considered). Finally, this study resulted in fewer positive outcomes, possibly related to only one follow-up examination and the unfavorable long-term course of T2D. In subsequent studies, our group will continue researching the association between leukocyte subtypes and T2D.

## 5 Conclusion

In the present study, elevated peripheral blood leukocyte counts and neutrophil levels were risk factors for developing T2D. In people with normal blood glucose at baseline, those with lower lifestyle scores were likelier to have a high peripheral blood leukocyte count. In addition to monitoring fasting blood glucose levels, the total number of leukocytes and their subgroup cell levels in the body should be monitored when working on T2D secondary and tertiary prevention.

## Data availability statement

The original contributions presented in the study are included in the article/**Supplementary Material**. Further inquiries can be directed to the corresponding author.

## Ethics statement

The studies involving human participants were reviewed and approved by The Ethics Committee of Xiangya School of Public Health, Central South University, China (No. XYGW-2016-10). The patients/participants provided their written informed consent to participate in this study.

## Author contributions

LL came up with the idea for the paper. RL, LL, and SX were responsible for the study design. RL was responsible for the data analyses and interpretation, manuscript writing, and revision. RL, BL, and LL were responsible for the data acquisition. RL and LL were responsible for the manuscript revision. DL was responsible for work arrangements at the cohort study sites. SX was responsible for the study conceptualization, data acquisition and interpretation, and manuscript revision. All of the authors contributed to the article and approved the submitted version.
