# Supplementary figures and images for: Associations of levels of peripheral blood leukocyte and subtypes with type 2 diabetes: A longitudinal study of Chinese government employees
Source: Front Endocrinol (Lausanne). 2023 Mar 24;14:1094022. doi: 10.3389/fendo.2023.1094022 (PMC10080122; doi:10.3389/fendo.2023.1094022)

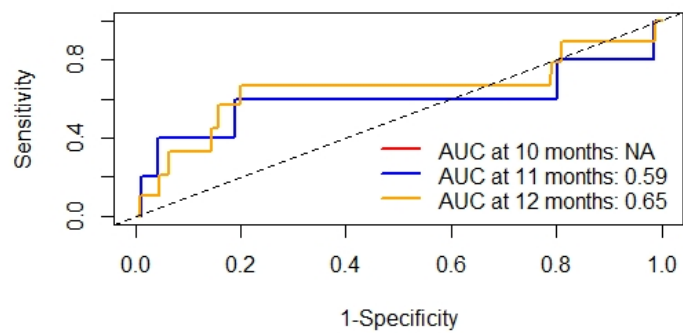

Leukocyte

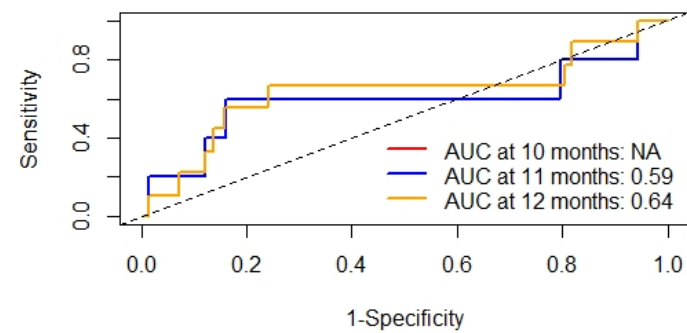

Neutrophil

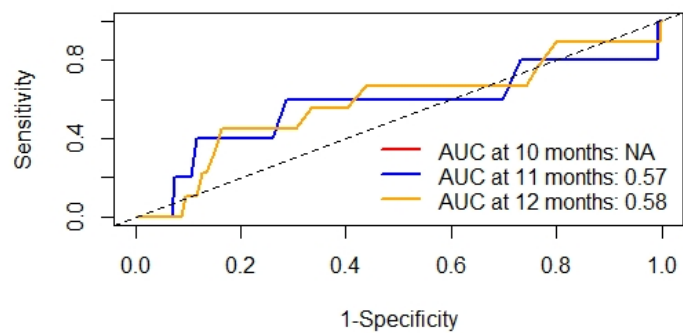

Monocyte

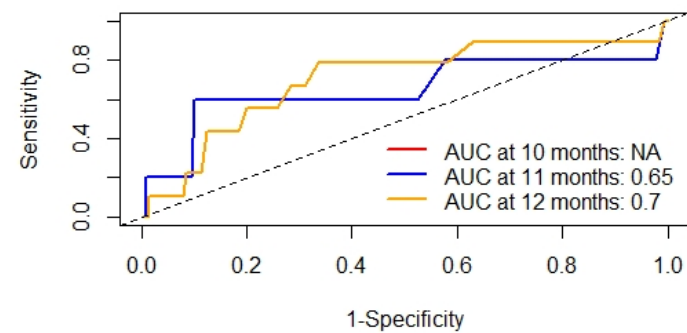

Eosinophil

Figure 1-A

Supplement: Supplementary file 1 [file Image_1.pdf]

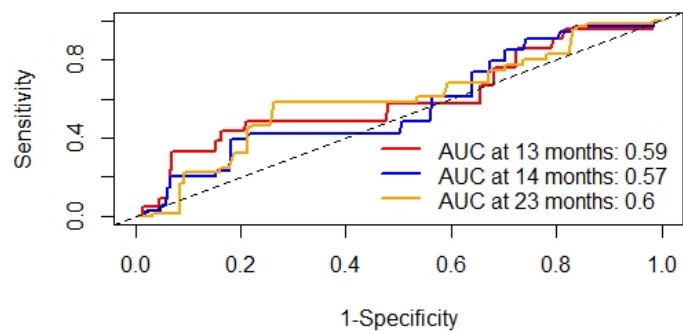

Leukocyte

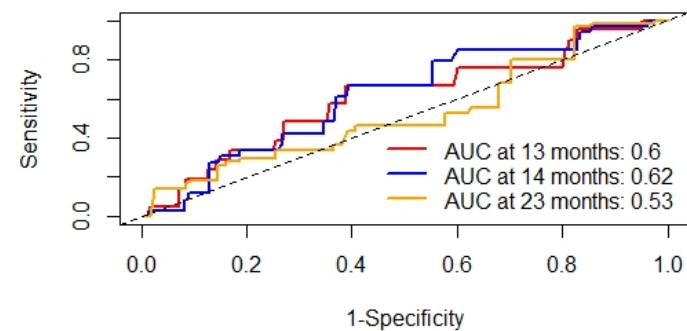

Neutrophil

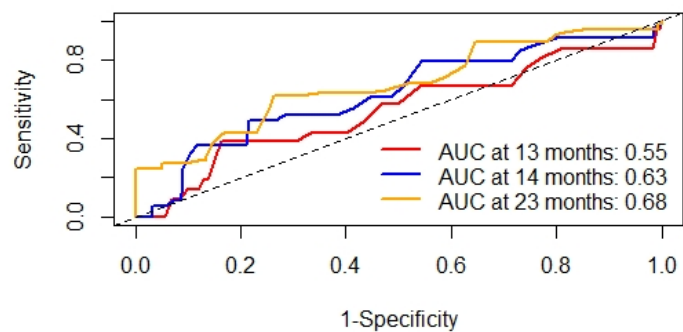

Monocyte

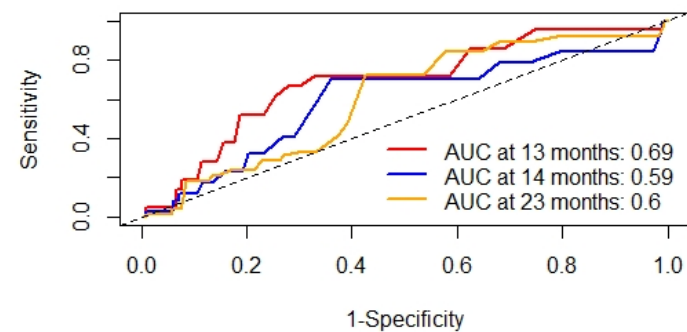

Eosinophil

Figure 1-B

Supplement: Supplementary file 2 [file Image_2.pdf]

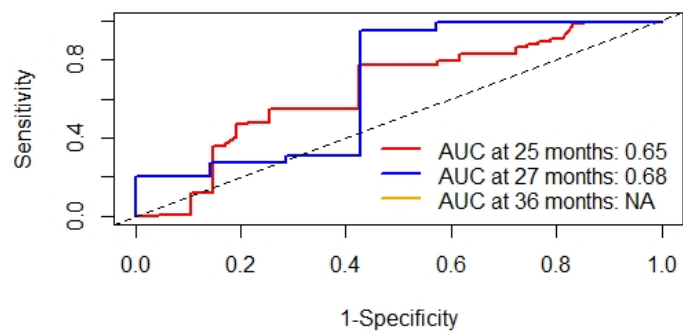

Leukocyte

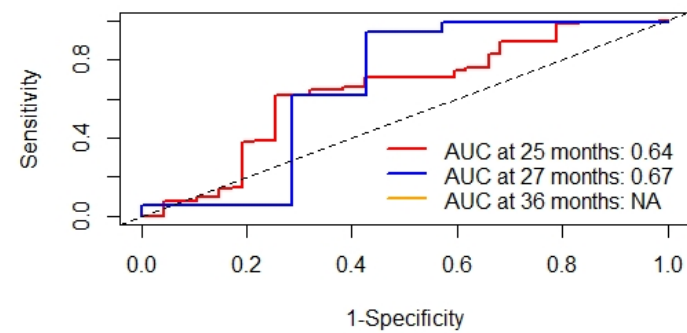

Neutrophil

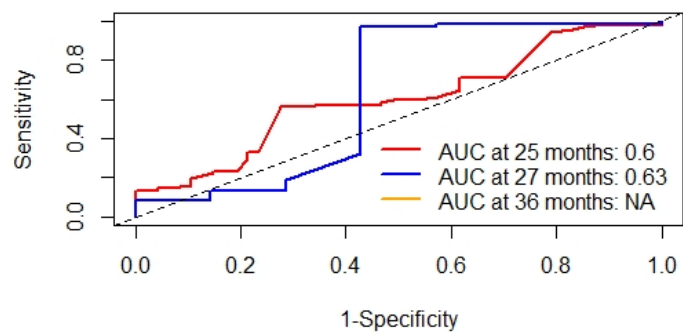

Monocyte

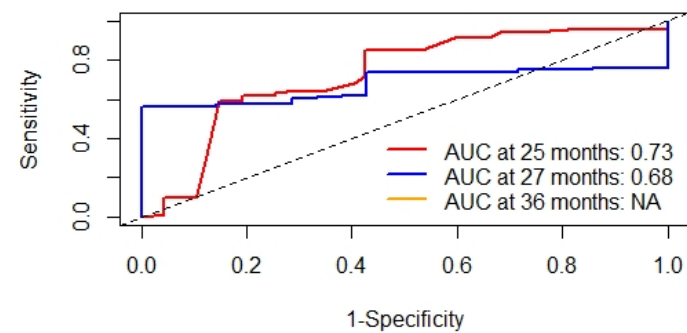

Eosinophil

Figure 1-C

Supplement: Supplementary file 3 [file Image_3.pdf]
