# Supplementary material for: Associations of levels of peripheral blood leukocyte and subtypes with type 2 diabetes: A longitudinal study of Chinese government employees
Source: Front Endocrinol (Lausanne). 2023 Mar 24;14:1094022. doi: 10.3389/fendo.2023.1094022 (PMC10080122; doi:10.3389/fendo.2023.1094022)
Supplement: Supplementary file 4 [file Table_1.docx]

Supplementary Material

**Table 1 Comparison of the basic characteristics of individuals excluded due to deletion and those not excluded**

| **Characteristics** | **Total**  **Population** | **Include in longitudinal analysis** | | ***P* value** |
| --- | --- | --- | --- | --- |
|  | **(n=10746)** | **Yes(n=5475)** | **No(n=5271)** |  |
| Location, n(%) |  |  |  | <0.001 |
| Huaihua | 487(4.5) | 361(74.1) | 126(25.9) |  |
| Changsha | 3611(33.6) | 2358(65.3) | 1253(34.7) |  |
| Zhuzhou | 3487(32.4) | 1243(35.6) | 2244(64.4) |  |
| Xiangtan | 3161(29.4) | 1513(47.9) | 1648(52.1) |  |
| Sex, n(%) |  |  |  | <0.001 |
| male | 3230(30.1) | 1560(48.3) | 1670(51.7) |  |
| female | 7516(69.9) | 3915(52.1) | 3601(47.9) |  |
| Age, median(IQR) | 33.10(12.50) | 33.00(11.80) | 34.00(13.90) | <0.001 |
| Education level, n(%) |  |  |  | <0.001 |
| High school or below | 357(4.3) | 221(61.9) | 136(38.1) |  |
| college | 5938(71.2) | 3825(64.4) | 2113(35.6) |  |
| Graduate or beyond | 2044(24.5) | 1429(69.9) | 615(30.1) |  |
| Marital, n(%) |  |  |  | <0.001 |
| Spinsterhood | 2139(19.9) | 1256(58.7) | 883(41.3) |  |
| Married | 7415(69.0) | 4101(55.3) | 3314(62.9) |  |
| Divorced or widowed | 238(2.2) | 114(47.9) | 124(52.1) |  |
| Others  NA | 9(0.1)  945(8.8) | 4(44.4)  0(0.0) | 5(55.6)  945(100.0) |  |
| leukocyte, median(IQR) | 6.15(2.00) | 6.10(2.00) | 6.24(2.00) | <0.001 |
| Lymphocyte, median(IQR) | 2.09(1.00) | 2.07(1.00) | 2.10(1.00) | 0.059 |
| Monocyte, median(IQR) | 0.34(0.00) | 0.34(0.00) | 0.35(0.00) | <0.001 |
| Neutrophil, median(IQR) | 3.47(2.00) | 3.43(2.00) | 3.53(2.00) | <0.001 |
| Eosinophil, median(IQR) | 0.11(0.00) | 0.11(0.00) | 0.11(0.00) | 0.069 |
| Basophil, median(IQR) | 0.02(0.00) | 0.02(0.00) | 0.02(0.00) | 0.001 |
| TC, median(IQR)^a^ | 4.52(1.13) | 4.49(1.12) | 4.58(1.14) | <0.001 |
| TG, median(IQR)^b^ | 1.01(0.87) | 0.97(0.82) | 1.08(0.92) | <0.001 |
| HDL, median(IQR)^c^ | 1.45(0.47) | 1.46(0.45) | 1.44(0.48) | 0.084 |
| LDL, median(IQR)^d^ | 2.59(0.96) | 2.60(0.96) | 2.59(0.98) | 0.414 |

^a^ Total cholesterol

^b^ Triglycerides

^c^ High-density lipoprotein

^d^ Low-density lipoprotein

**Table 2 Comparison of baseline characteristics between new cases, non-cases, and unknown ending**

| **Characteristics** | **Total**  **Population** | **New cases** | | | ***P* value** |
| --- | --- | --- | --- | --- | --- |
|  | **(n=5013)** | **Yes** | **No** | **NA** |  |
| Location, n(%) |  |  |  |  | <0.001 |
| Huaihua | 286(5.7) | 4(1.4) | 239(83.6) | 43(15.0) |  |
| Changsha | 2244(44.8) | 19(0.8) | 2216(98.8) | 9(0.4) |  |
| Zhuzhou | 1106(22.1) | 3(0.3) | 988(89.3) | 115(10.4) |  |
| Xiangtan | 1377(27.5) | 3(0.2) | 1186(86.1) | 188(13.7) |  |
| Sex, n(%) |  |  |  |  | <0.001 |
| male | 1318(26.3) | 22(1.7) | 1135(86.1) | 161(12.2) |  |
| female | 3695(73.7) | 3494(94.6) | 7(0.2) | 194(5.3) |  |
| Age, median(IQR) | 33.00(11.20) | 41.00(16.00) | 33.00(10.90) | 33.10(11.60) | <0.001 |
| Education level, n(%) |  |  |  |  | 0.034 |
| High school or below | 181(3.6) | 2(1.1) | 175(96.7) | 4(2.2) |  |
| college | 3526(70.3) | 17(0.5) | 3243(92.0) | 266(7.5) |  |
| Graduate or beyond | 1306(26.1) | 10(0.8) | 1211(92.7) | 85(6.5) |  |
| Marital, n(%) |  |  |  |  | 0.025 |
| Spinsterhood | 1124(22.4) | 2(0.2) | 1038(92.3) | 84(7.5) |  |
| Married | 3789(75.6) | 24(0.6) | 3504(92.5) | 261(6.9) |  |
| Divorced or widowed | 96(1.9) | 3(3.1) | 83(86.5) | 10(10.4) |  |
| others | 4(0.1) | 0(0.00) | 4(100.00) | 0(0.0) |  |
| Family history of diabetes mellitus, n(%) |  |  |  |  | 0.038 |
| Yes | 787(15.7) | 6(0.8) | 709(90.1) | 72(9.1) |  |
| No | 4226(84.3) | 3920(92.8) | 23(0.5) | 283(6.7) |  |
| Smoke status, n(%) |  |  |  |  | <0.001 |
| Current smoker | 463(9.2) | 11(2.4) | 401(86.6) | 51(11.0) |  |
| Former smoker | 55(1.1) | 0(0.0) | 49(89.1) | 6(10.9) |  |
| Never smoker | 4290(85.6) | 17(0.4) | 3995(93.1) | 278(6.5) |  |
| Passive smoking | 205(4.1) | 1(0.5) | 184(89.8) | 20(9.8) |  |
| Drinking status, n(%) |  |  |  |  | <0.001 |
| Current drinker | 671(13.4) | 7(1.0) | 588(87.6) | 76(11.3) |  |
| Former drinker | 27(0.5) | 2(7.4) | 23(85.2) | 2(7.4) |  |
| Never drinker | 4315(86.1) | 20(0.5) | 4018(93.1) | 277(6.4) |  |
| Exercise, n(%) |  |  |  |  | 0.100 |
| Yes | 2306(46.0) | 17(0.7) | 2111(91.5) | 178(7.7) |  |
| No | 2707(54.0) | 12(0.4) | 2518(93.0) | 177(6.5) |  |
| Body shape, n(%) |  |  |  |  | <0.001 |
| No.1 shape^a^ | 3876(77.3) | 11(0.3) | 3601(92.9) | 264(6.8) |  |
| No.2 shape^b^ | 390(7.8) | 0(0.0) | 366(93.8) | 24(6.2) |  |
| No.3 shape^c^ | 348(6.9) | 8(2.3) | 309(88.8) | 31(8.9) |  |
| No.4 shape^d^ | 138(2.8) | 0(0.0) | 132(95.7) | 6(4.3) |  |
| No.5 shape^e^ | 261(5.2) | 10(3.8) | 221(84.7) | 30(11.5) |  |

**Continued Table 2.1**

| Diet score, n(%) |  |  |  |  | 0.436 |
| --- | --- | --- | --- | --- | --- |
| 0 | 274(5.5) | 0(0.0) | 254(92.7) | 20(7.3) |  |
| 1 | 1142(22.8) | 5(0.4) | 1064(93.2) | 73(6.4) |  |
| 2 | 1555(31.0) | 8(0.5) | 1429(91.9) | 8(0.5) |  |
| 3 | 1238(24.7) | 8(0.6) | 1145(92.5) | 85(6.9) |  |
| 4 | 694(13.8) | 8(1.2) | 632(91.1) | 54(7.8) |  |
| 5 | 110(2.2) | 0(0.0) | 105(95.5) | 5(4.5) |  |
| Lifestyle score, n(%) |  |  |  |  | 0.001 |
| 0 | 56(1.1) | 1(1.8) | 47(83.9) | 8(14.3) |  |
| 1 | 243(4.8) | 5(2.1) | 212(87.2) | 26(10.7) |  |
| 2 | 856(17.1) | 6(0.7) | 779(91.0) | 71(8.3) |  |
| 3 | 2099(41.9) | 13(0.6) | 1951(92.9) | 135(6.4) |  |
| 4 | 1456(29.0) | 3(0.2) | 1361(93.5) | 92(6.3) |  |
| 5 | 303(6.0) | 1(0.3) | 279(92.1) | 23(7.6) |  |
| leukocyte, median(IQR) | 6.06(2.06) | 7.23(2.46) | 6.05(2.01) | 6.29(2.27) | 0.001 |
| Lymphocyte, median(IQR) | 2.06(0.80) | 2.04(0.96) | 2.05(0.79) | 2.20(0.90) | <0.001 |
| Monocyte, median(IQR) | 0.33(0.15) | 0.39(0.19) | 0.33(0.15) | 0.36(0.17) | <0.001 |
| Neutrophil, median(IQR) | 3.40(1.48) | 3.90(1.69) | 3.40(1.48) | 3.41(1.58) | 0.032 |
| Eosinophil, median(IQR) | 0.11(0.11) | 0.18(0.16) | 0.10(0.11) | 0.12(0.13) | <0.001 |
| Basophil, median(IQR) | 0.02(0.02) | 0.03(0.03) | 0.02(0.02) | 0.02(0.02) | 0.057 |
| TG, median(IQR)^e^ | 0.95(0.78) | 1.77(1.63) | 0.94(0.74) | 1.16(1.23) | <0.001 |

^a^ body shape 1: BMI 18.5-27.9(kg/m^2^), waist circumference < 90 cm of men or < 85 cm of women

^b^ body shape 2:BMI<18.5(kg/m^2^)

^c^ body shape 3: BMI 18.5-27.9(kg/m^2^), waist circumference ≥ 90 cm of men or ≥ 85 cm of women

^d^ body shape 4: BMI ≥ 28.0(kg/m^2^), waist circumference < 90 cm of men or < 85 cm of women

^e^ body shape 5: BMI ≥ 28.0(kg/m^2^), waist circumference ≥ 90 cm of men or ≥ 85 cm of women

^e^ Triglycerides

**Table 3 Comparison of baseline characteristics between with and without follow-up endings**

| **Characteristics** | **Total**  **Population** | **Follow-up endings** | | ***P* value** |
| --- | --- | --- | --- | --- |
|  | **(n=5013)** | **Yes(n=4658)** | **No(n=355)** |  |
| Location, n(%) |  |  |  | <0.001 |
| Huaihua | 286(5.7) | 243(85.0) | 43(15.0) |  |
| Changsha | 2244(44.8) | 2235(99.6) | 9(0.4) |  |
| Zhuzhou | 1106(22.1) | 991(89.6) | 115(10.4) |  |
| Xiangtan | 1377(27.5) | 1189(86.3) | 188(13.7) |  |
| Sex, n(%) |  |  |  | <0.001 |
| male | 1318(26.3) | 1157(87.8) | 161(12.2) |  |
| female | 3695(73.7) | 3501(94.7) | 194(5.3) |  |
| Age, median(IQR) | 33.00(11.20) | 33.00(11.03) | 33.10(11.60) | 0.534 |
| Education level, n(%) |  |  |  | 0.016 |
| High school or below | 181(3.6) | 177(97.8) | 4(2.2) |  |
| college | 3526(70.3) | 3260(92.5) | 266(7.5) |  |
| Graduate or beyond | 1306(26.1) | 1221(93.5) | 85(6.5) |  |
| Marital, n(%) |  |  |  | 0.014 |
| Spinsterhood | 2139(19.9) | 1256(58.7) | 883(41.3) |  |
| Married | 7415(69.0) | 4101(55.3) | 3314(62.9) |  |
| Divorced or widowed | 238(2.2) | 114(47.9) | 124(52.1) |  |
| Others  NA | 9(0.1) | 4() | 5(55.6) |  |
| Family history of diabetes mellitus, n(%) |  |  |  | 0.014 |
| Yes | 787(15.7) | 715(90.9) | 72(9.1) |  |
| No | 4226(84.3) | 3943(93.3) | 283(6.7) |  |
| Smoke status, n(%) |  |  |  | 0.001 |
| Current smoker | 463(9.2) | 412(8.8) | 51(11.0) |  |
| Former smoker | 55(1.1) | 49(89.1) | 6(10.9) |  |
| Never smoker | 4290(85.6) | 4012(93.5) | 278(6.5) |  |
| Passive smoking | 205(4.1) | 185(90.2) | 20(9.8) |  |
| Drinking status, n(%) |  |  |  | <0.001 |
| Current drinker | 671(13.4) | 595(88.7) | 76(11.3) |  |
| Former drinker | 27(0.5) | 25(92.6) | 2(7.4) |  |
| Never drinker | 4315(86.1) | 4038(93.6) | 277(6.4) |  |
| Exercise, n(%) |  |  |  | 0.104 |
| Yes | 2306(46.0) | 2128(92.3) | 178(7.7) |  |
| No | 2707(54.0) | 2530(93.5) | 177(6.5) |  |
| Body shape, n(%) |  |  |  | 0.017 |
| No.1 shape^a^ | 3876(77.3) | 3612(93.2) | 264(6.8) |  |
| No.2 shape^b^ | 390(7.8) | 366(93.8) | 24(6.2) |  |
| No.3 shape^c^ | 348(6.9) | 317(91.1) | 31(8.9) |  |
| No.4 shape^d^ | 138(2.8) | 132(95.7) | 6(4.3) |  |
| No.5 shape^e^ | 261(5.2) | 231(88.5) | 30(18.5) |  |

**Continued Table 3.1**

| Diet score, n(%) |  |  |  | 0.680 |
| --- | --- | --- | --- | --- |
| 0 | 274(5.5) | 254(92.7) | 20(7.3) |  |
| 1 | 1142(22.8) | 1069(93.6) | 73(6.4) |  |
| 2 | 1555(31.0) | 1437(92.4) | 118(7.6) |  |
| 3 | 1238(24.7) | 1153(93.1) | 85(6.9) |  |
| 4 | 694(13.8) | 640(92.2) | 54(7.8) |  |
| 5 | 110(2.2) | 105(95.5) | 5(4.5) |  |
| Lifestyle score, n(%) |  |  |  | 0.016 |
| 0 | 56(1.1) | 48(85.7) | 8(14.3) |  |
| 1 | 243(4.8) | 217(89.3) | 26(10.7) |  |
| 2 | 856(17.1) | 785(91.7) | 71(8.3) |  |
| 3 | 2099(41.9) | 1964(93.6) | 135(6.4) |  |
| 4 | 1456(29.0) | 1364(93.7) | 92(25.9) |  |
| 5 | 303(6.0) | 280(92.4) | 23(7.6) |  |
| leukocyte, median(IQR) | 6.06(2.06) | 6.05(2.02) | 6.29(2.27) | 0.007 |
| Lymphocyte, median(IQR) | 2.06(0.80) | 2.05(0.79) | 2.20(0.90) | <0.001 |
| Monocyte, median(IQR) | 0.33(0.15) | 0.33(0.15) | 0.36(0.17) | <0.001 |
| Neutrophil, median(IQR) | 3.40(1.48) | 3.40(1.47) | 3.41(1.58) | 0.356 |
| Eosinophil, median(IQR) | 0.11(0.11) | 0.10(0.11) | 0.12(0.13) | 0.001 |
| Basophil, median(IQR) | 0.02(0.02) | 0.02(0.02) | 0.02(0.02) | 0.052 |
| TG, median(IQR)^e^ | 0.95(0.78) | 0.94(0.75) | 1.16(1.23) | <0.001 |

^a^ body shape 1: BMI 18.5-27.9(kg/m^2^), waist circumference < 90 cm of men or < 85 cm of women

^b^ body shape 2:BMI<18.5(kg/m^2^)

^c^ body shape 3: BMI 18.5-27.9(kg/m^2^), waist circumference ≥ 90 cm of men or ≥ 85 cm of women

^d^ body shape 4: BMI ≥ 28.0(kg/m^2^), waist circumference < 90 cm of men or < 85 cm of women

^e^ body shape 5: BMI ≥ 28.0(kg/m^2^), waist circumference ≥ 90 cm of men or ≥ 85 cm of women

^e^ Triglycerides

**Figure Legends**

Supplementary figure 1-A. Time-dependent ROC curves for the subtype of leukocytes (months: 10, 11, 12)

Supplementary figure 1-B. Time-dependent ROC curves for the subtype of leukocytes (months: 13, 14, 23)

Supplementary figure 1-C. Time-dependent ROC curves for the subtype of leukocytes (months: 25, 27, 36)
